# Supplementary material for: Temporary Consolidation of Marine Artifact Based on Polyvinyl Alcohol/Tannic Acid Reversible Hydrogel
Source: Polymers (Basel). 2023 Dec 5;15(24):4621. doi: 10.3390/polym15244621 (PMC10747287; doi:10.3390/polym15244621)
Supplement: Supplementary file 1 [file polymers-15-04621-s001.zip › polymers-2715948-supplementary/Supplementary/20231028supplementary.pdf]

Figure S1 describes the result of detached wood samples revealed that hydrogel had no impact on the wood samples. Table S1 show that B and Ca had a little residue on the surface of the wood samples.

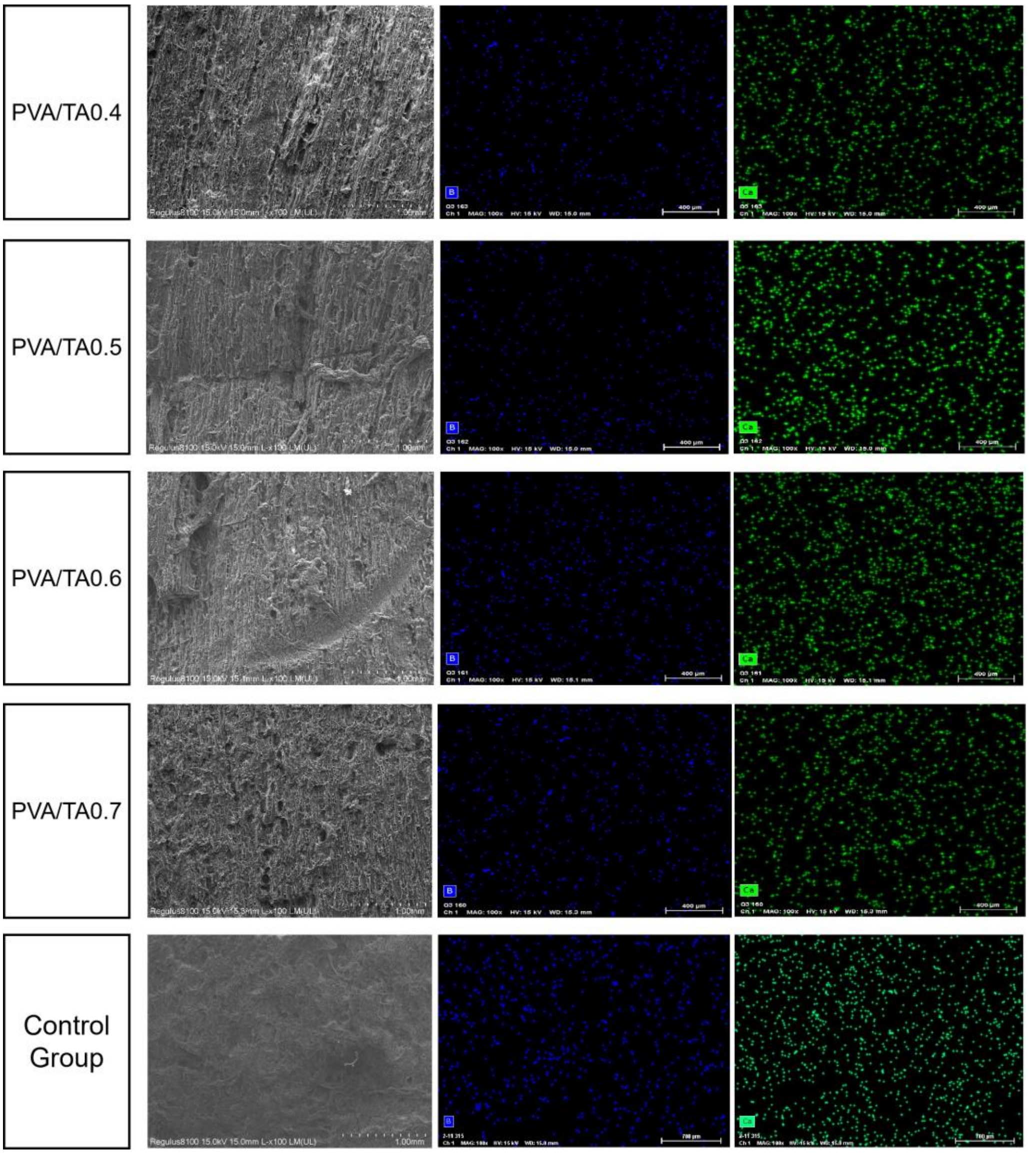

Figure S1: SEM images of wood samples after hydrogel oxidated and surface scanning images of B and Ca elements

Table S1: EDS result of B and Ca element in the wood samples surface (%)

Element	PVA/TA0.4	PVA/TA0.5	PVA/TA0.6	PVA/TA0.7	Control Group
B	1.30	1.48	1.68	0.8	0.26
Ca	0.77	0.75	0.91	0.26	0.21

Figure S2 shows that using Na2EDTA immersion to removal the hydrogel had no impact on the wood sample, and Table S2 indicate that the B content on the wood surface after hydrogel removal remained almost unchanged when the hydrogels were removed.

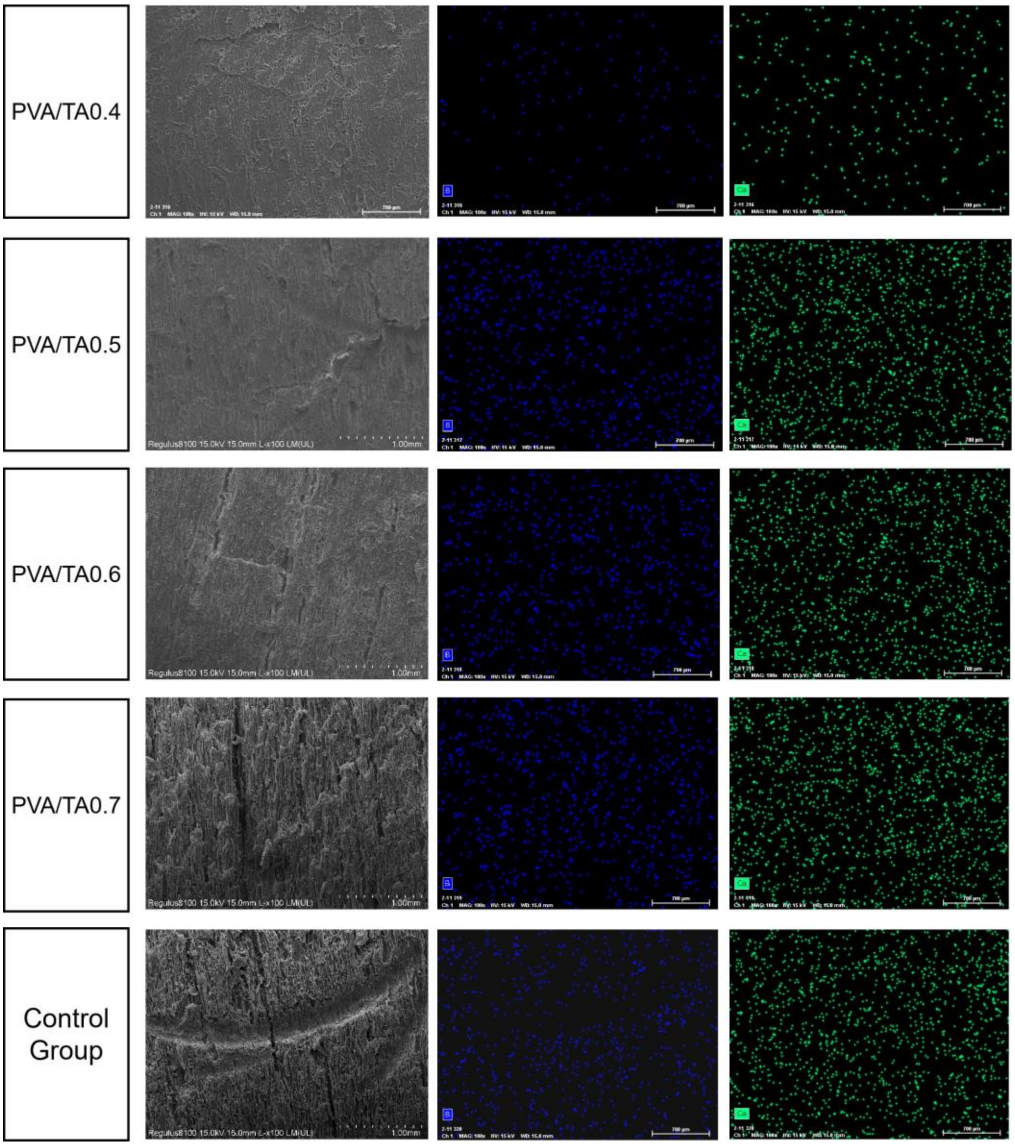

Figure S2: SEM images of wood samples after hydrogel removed by immersion in Na2EDTA solution and surface scanning images of B and Ca elements

Table S2: EDS result of B and Ca element in the wood sample surface (%)

Element	PVA/TA0.4	PVA/TA0.5	PVA/TA0.6	PVA/TA0.7	Control Group
B	1.41	1.25	2.09	0.95	1.16
Ca	0.21	0.28	0.23	0.26	0.08

Figure S3 and Table S3 indicate the PVA/TA0.4, PVA/TA0.5, PVA/TA0.6 and PVA/TA0.7 samples showed an increase in the content of B and Ca, as well as a reduction in the content of Fe.

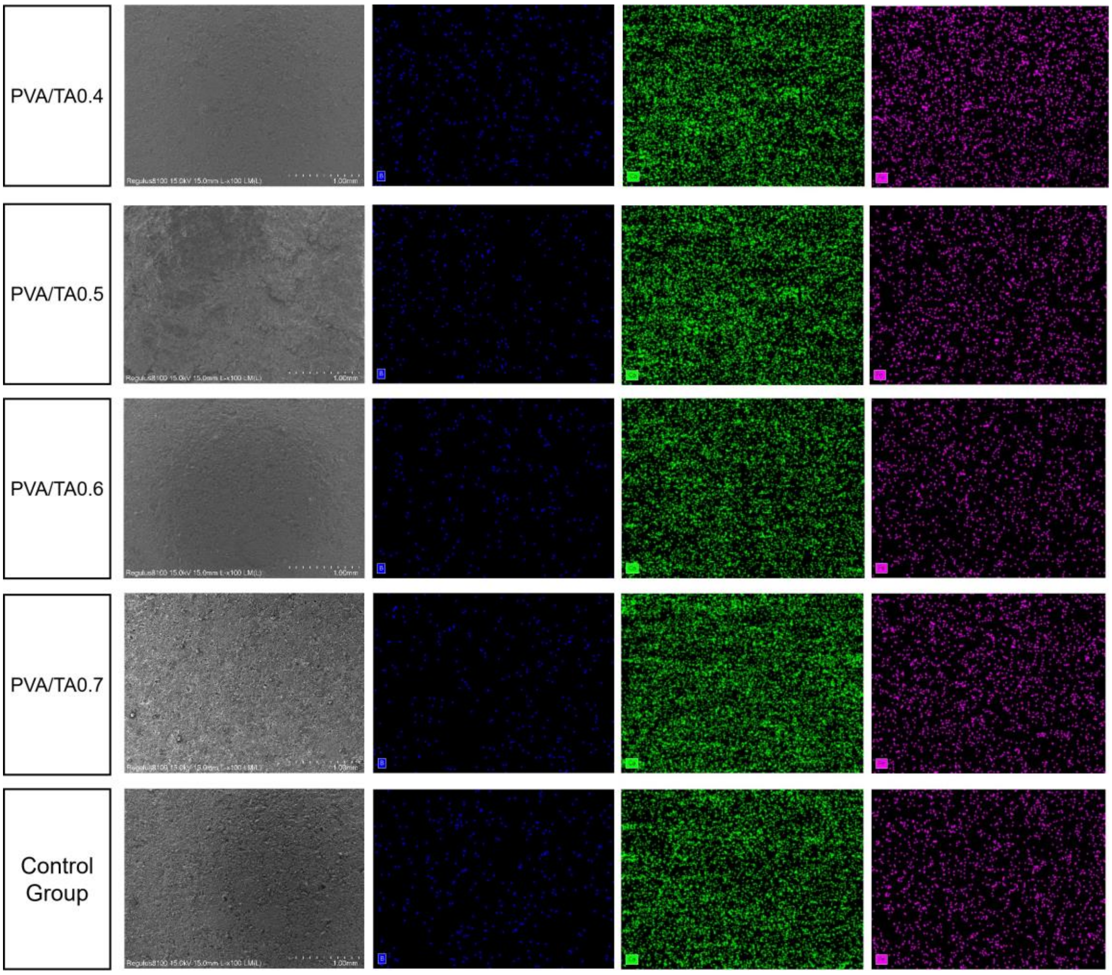

Figure S3: SEM images of pottery samples after hydrogel oxidated and surface scanning images of B, Ca and Fe elements

Table S3: EDS result of B, Ca and Fe element in the pottery samples surface (%)

Element	PVA/TA0.4	PVA/TA0.5	PVA/TA0.6	PVA/TA0.7	Control Group
B	4.10	4.21	4.47	4.18	3.97
Ca	6.83	6.09	5.21	5.95	5.01
Fe	2.39	2.50	1.95	1.62	2.69

Table S4 indicate that the black substance should consist of a hydrogel complexed with iron ions.

Table S4 EDS result of the black substance

Element	B	C	O	Ca	Fe
Content(%)	2.67	63.1	32.23	1.34	0.63

Table S4 indicate that the four groups of samples exhibited a significant increase in the B element and a decrease in the Ca and Fe after hydrogel removed by immersion in a Na_2EDTA solution.

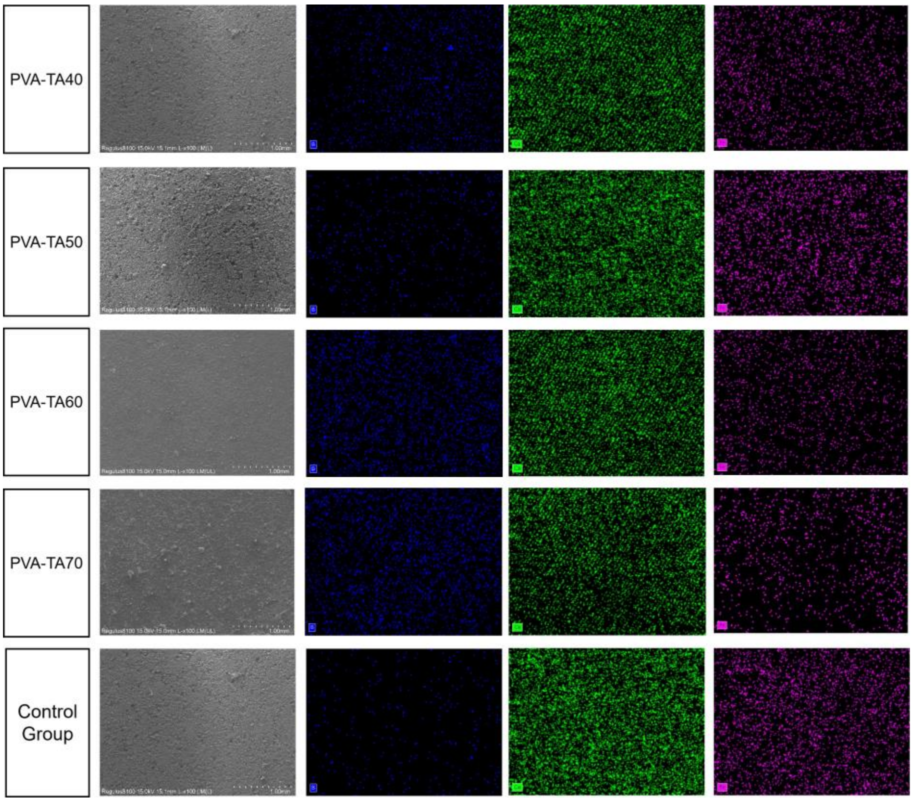

Figure S4: SEM images of pottery samples after hydrogel removed by immersion in Na2EDTA solution and surface scanning images of B, Ca and Fe elements

Table S5: EDS result of B, Ca and Fe element in the pottery sample surface

Element	PVA/TA0.4	PVA/TA0.5	PVA/TA0.6	PVA/TA0.7	Control Group
B	4.55	4.92	6.34	5.51	3.33
Ca	5.06	4.77	3.57	3.59	5.51
Fe	2.49	1.92	0.88	1.51	3.67
