# Supplementary figures and images for: Temporary Consolidation of Marine Artifact Based on Polyvinyl Alcohol/Tannic Acid Reversible Hydrogel
Source: Polymers (Basel). 2023 Dec 5;15(24):4621. doi: 10.3390/polym15244621 (PMC10747287; doi:10.3390/polym15244621)

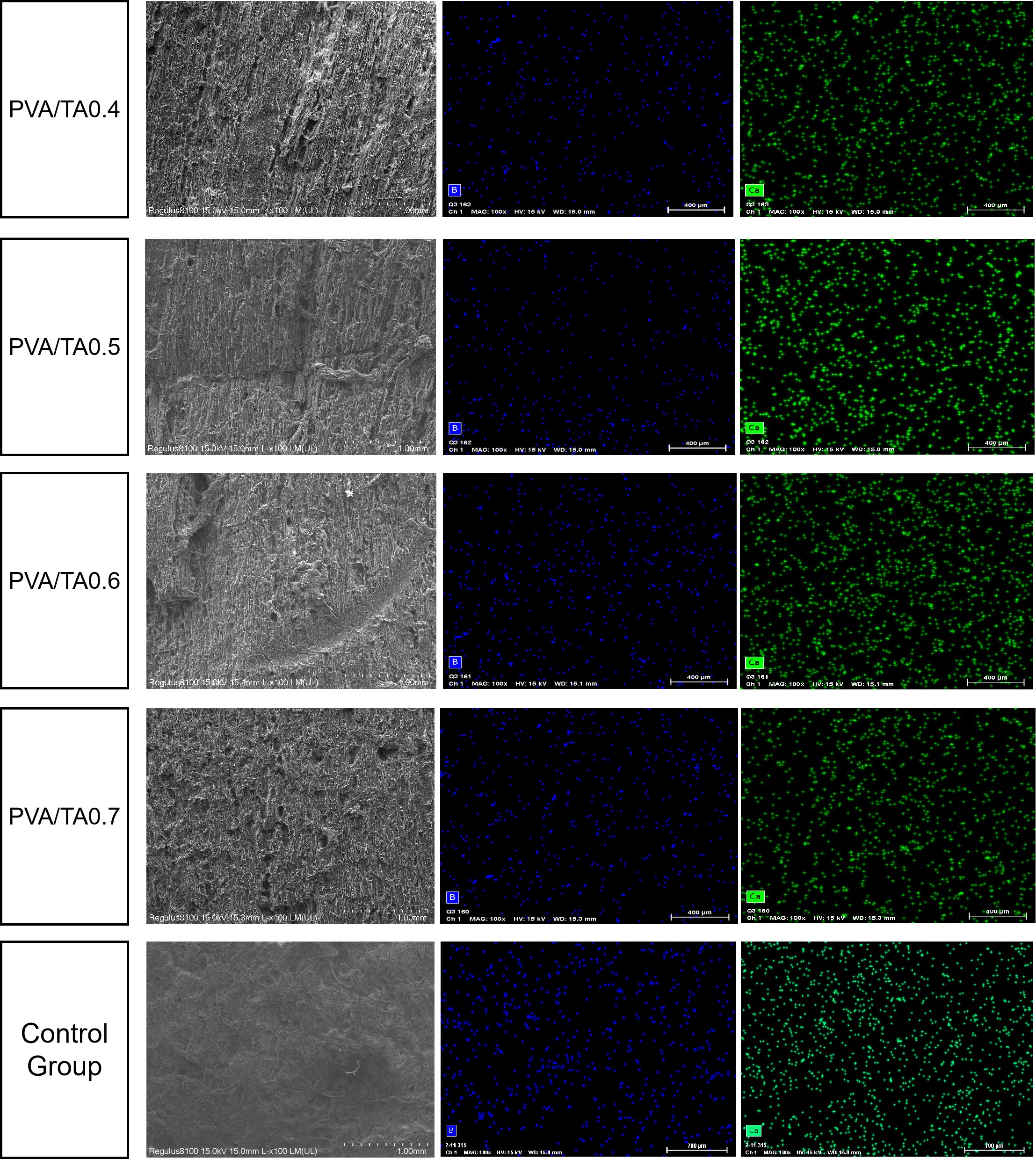

Supplement: Supplementary file 1 [file polymers-15-04621-s001.zip › polymers-2715948-supplementary/Supplementary/Figure S1.jpg]

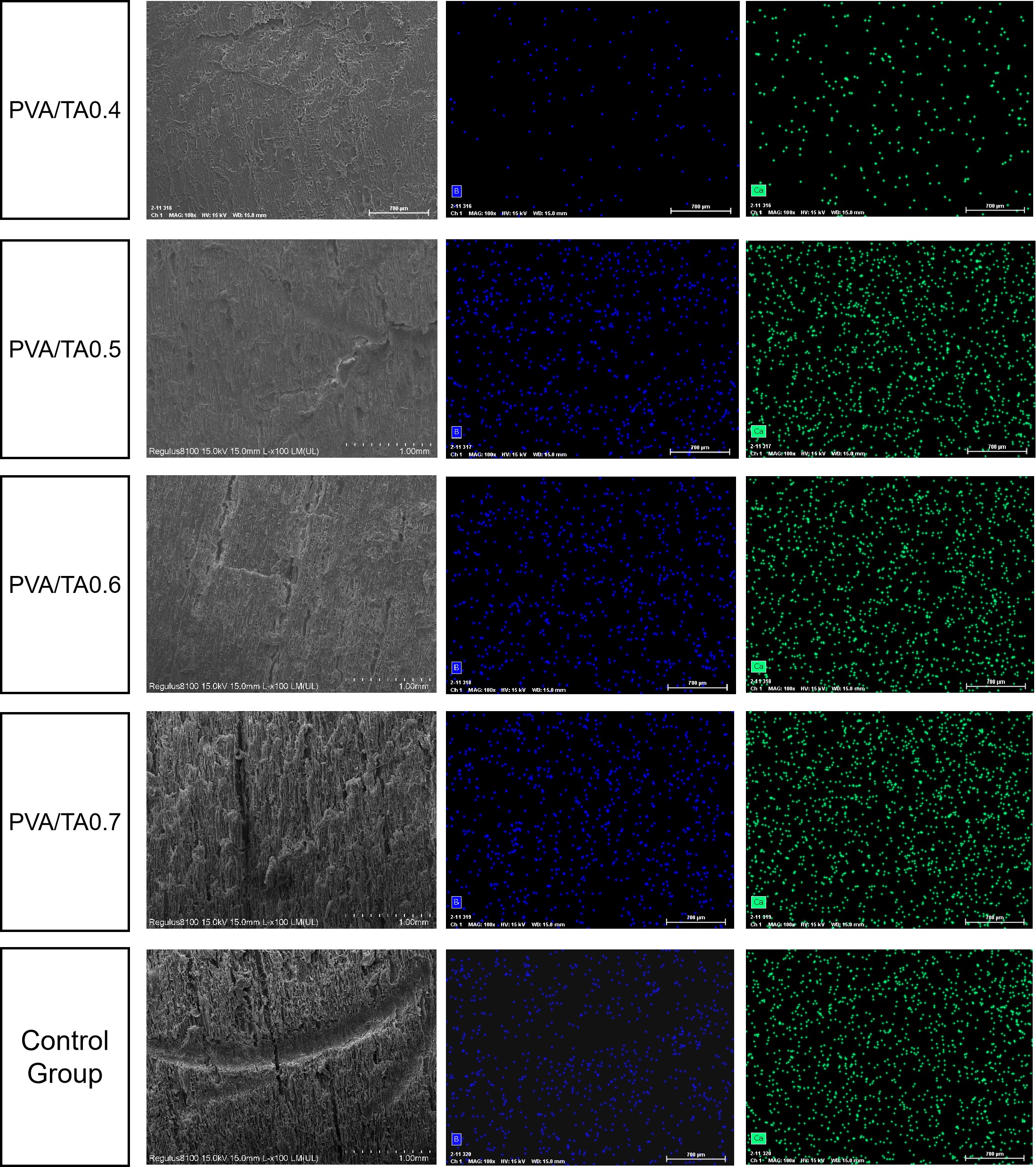

Supplement: Supplementary file 1 [file polymers-15-04621-s001.zip › polymers-2715948-supplementary/Supplementary/Figure S2.jpg]

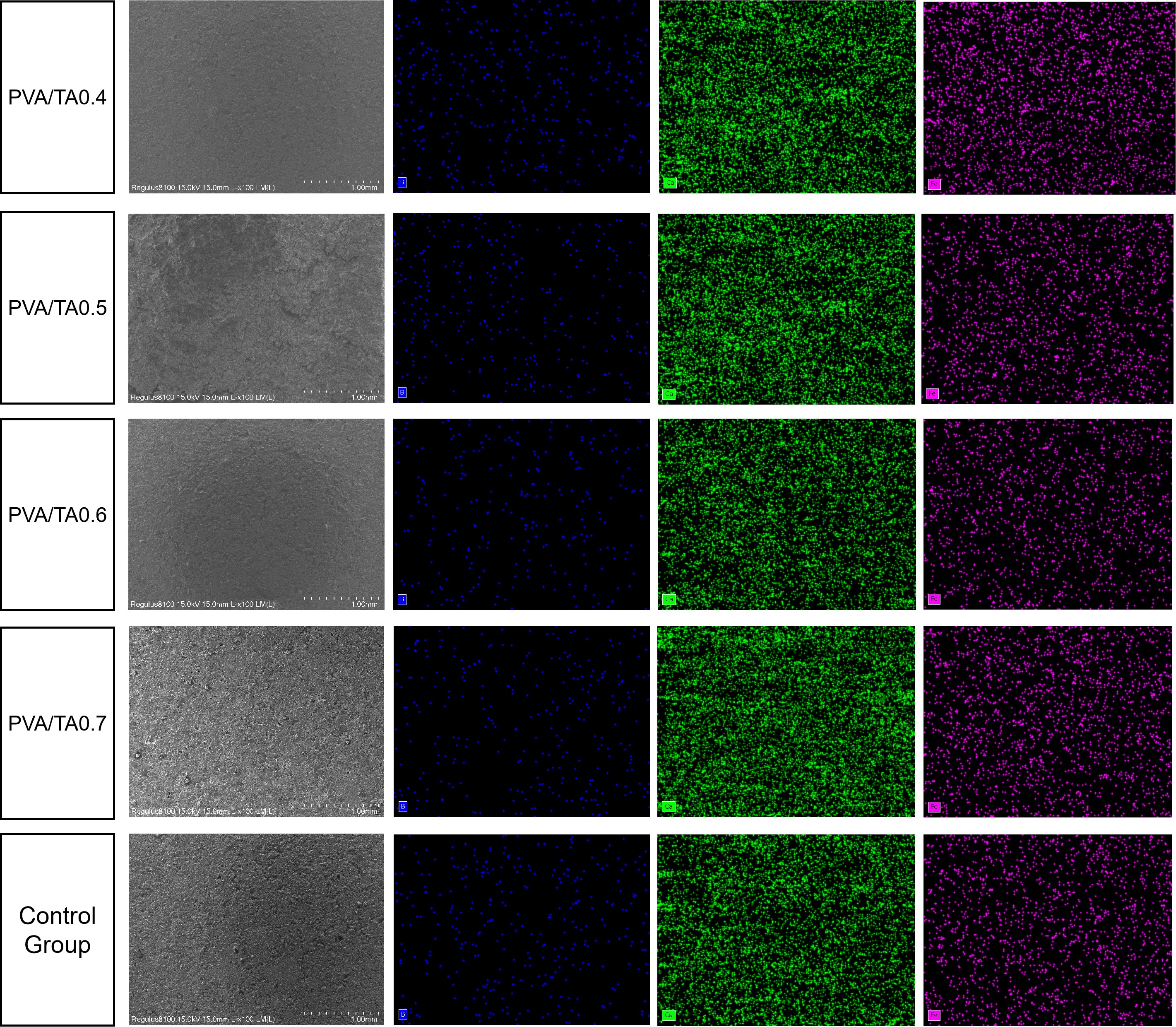

Supplement: Supplementary file 1 [file polymers-15-04621-s001.zip › polymers-2715948-supplementary/Supplementary/Figure S3.jpg]

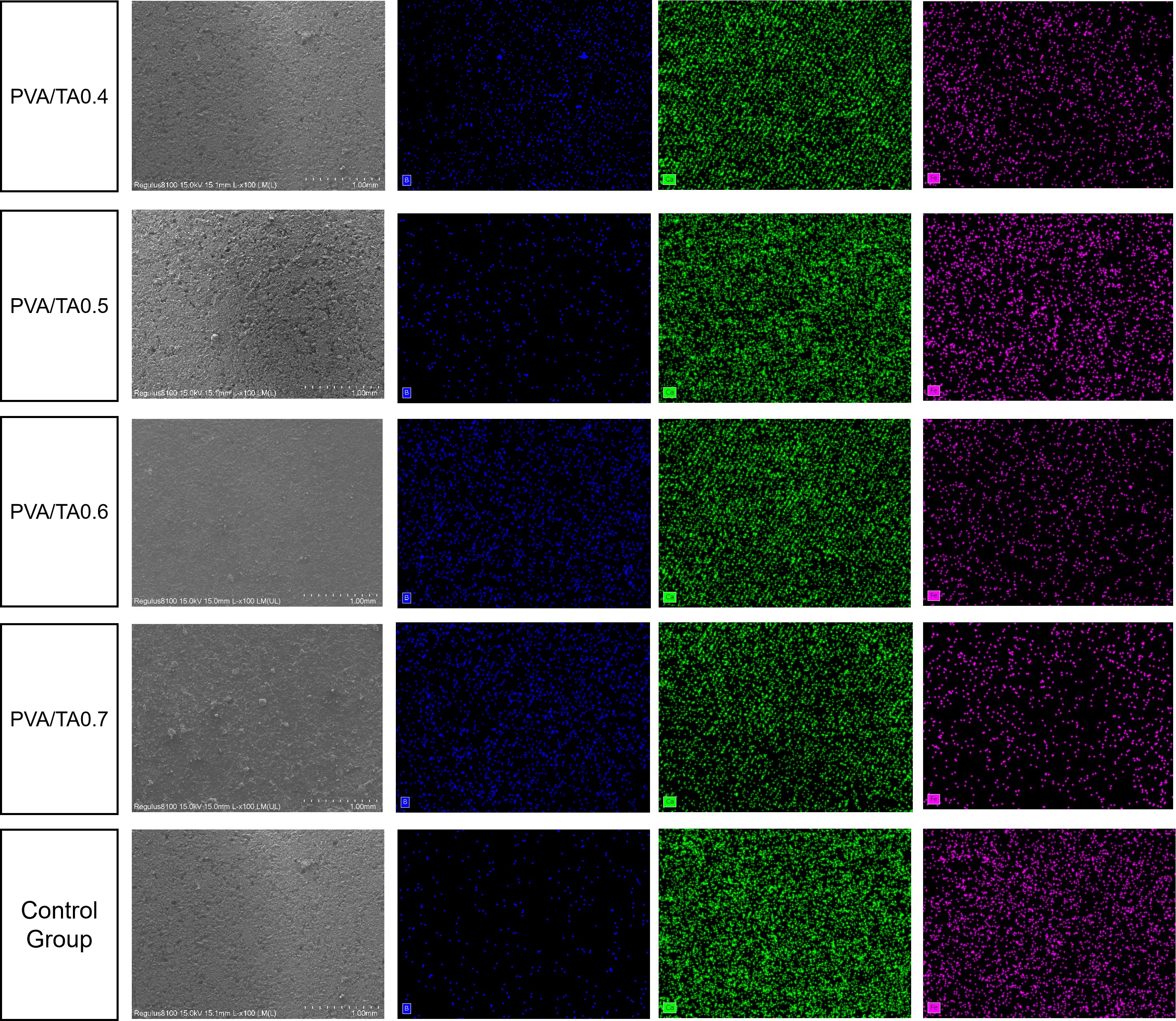

Supplement: Supplementary file 1 [file polymers-15-04621-s001.zip › polymers-2715948-supplementary/Supplementary/Figure S4.jpg]
